# Modification and Characterization of Fe_3_O_4_ Nanoparticles for Use in Adsorption of Alkaloids

**DOI:** 10.3390/molecules23030562

**Published:** 2018-03-02

**Authors:** Linyan Yang, Jing Tian, Jiali Meng, Ruili Zhao, Cun Li, Jifei Ma, Tianming Jin

**Affiliations:** 1 College of Animal Science and Veterinary Medicine, Tianjin Agricultural University, Tianjin 300384, China; y_linyan@163.com (L.Y.); 15822858982@163.com (J.T.); mjl19930123@163.com (J.M.); zhaoruili1109@126.com (R.Z.); 2 Guangxi Key Laboratory for the Chemistry and Molecular Engineering of Medicinal Resources, Chemical and Pharmaceutical College of Guangxi Normal University, Guilin 541004, China

**Keywords:** Fe_3_O_4_, modification, alginate, alkaloid

## Abstract

Magnetite (Fe_3_O_4_) is a ferromagnetic iron oxide of both Fe(II) and Fe(III), prepared by FeCl_2_ and FeCl_3_. XRD was used for the confirmation of Fe_3_O_4_. Via the modification of Tetraethyl orthosilicate (TEOS), (3-Aminopropyl)trimethoxysilane (APTMS), and Alginate (AA), Fe_3_O_4_@SiO_2_, Fe_3_O_4_@SiO_2_-NH_2_, and Fe_3_O_4_@SiO_2_-NH_2_-AA nanoparticles could be obtained, and IR and SEM were used for the characterizations. Alkaloid adsorption experiments exhibited that, as for Palmatine and Berberine, the most adsorption could be obtained at pH 8 when the adsorption time was 6 min. The adsorption percentage of Palmatine was 22.2%, and the adsorption percentage of Berberine was 23.6% at pH 8. Considering the effect of adsorption time on liquid phase system, the adsorption conditions of 8 min has been chosen when pH 7 was used. The adsorption percentage of Palmatine was 8.67%, and the adsorption percentage of Berberine was 7.25%. Considering the above conditions, pH 8 and the adsorption time of 8min could be chosen for further uses.

## 1. Introduction

Although there are many pure phases of iron oxide in nature, the most popular magnetic nanoparticles (MNPs) are the nanoscale zero-valent iron (nZVI), Fe_3_O_4_ and γ-Fe_2_O_3_. Magnetite (Fe_3_O_4_) is a ferromagnetic black color iron oxide of both Fe(II) and Fe(III), which has been the most extensively studied [1]. In 2001, Asher reported co-precipitation method using oleic acid as the surface modification agent to obtain Fe_3_O_4_ nanoparticles (2–15 nm) [2]. NaOH and diethylene glycol could also be used as the catalyst and reducing agent to fabricate Fe_3_O_4_ nanoparticles of 80–180 nm in size [3,4,5]. However, Fe_3_O_4_ nanoparticles could easily aggregate due to the nanoscale effect and magnetic gravitational effect. It is an effective method of preventing the aggregate of these nanoparticles to wrap the surface of Fe_3_O_4_ nanoparticles. Fe_3_O_4_@SiO_2_ composite nanoparticles have the desirable properties of magnetic nanoparticles while also benefiting from the SiO_2_ shell, such as good hydrophilicity, stability, and biocompatibility [6,7,8]. In 2016, Tang reported that (3-aminopropyl)-triethoxysilane (APTES) was used as surface modification reagents to get Fe_3_O_4_@SiO_2_-NH_2_, which could be used for selective removal of Zn(II) ions from wastewater [9]. While Fe_3_O_4_@SiO_2_-NH_2_ nanoparticles could also be modified to obtain mercaptoamine-functionalised silica-coated magnetic nanoparticles for the removal of mercury and lead ions from wastewater [10]. As for the removal of ions, arsenate removal could be achieved by calcium alginate-encapsulated magnetic sorbent, which was prepared by physical method [11]. Superparamagnetic sodium alginate-coated Fe_3_O_4_ nanoparticles (Alg-Fe_3_O_4_) were used for removal of malachite green (MG) from aqueous solutions using batch adsorption technique, and the Alg-Fe_3_O_4_ nanoparticles were synthesized using in situ coprecipitation of FeCl_2_ and FeCl_3_ in alkaline solution in the presence of sodium alginate [12]. While multifunctional alginate microspheres could also be used for biosensing, drug delivery, and magnetic resonance imaging [13]. To obtain the good biocompatibility, Fe_3_O_4_ nanoparticles need to be modified. Fe_3_O_4_@SiO_2_ composite nanoparticles have the desirable properties of good hydrophilicity. (3-Aminopropyl)trimethoxysilane (APTMS) was used as surface modification reagents to get Fe_3_O_4_@SiO_2_-NH_2_nanoparticles. While calcium alginate-encapsulated magnetic sorbent could be prepared by physical method. Superparamagnetic sodium alginate-coated Fe_3_O_4_ nanoparticles (Alg-Fe_3_O_4_) could also be synthesized using in situ coprecipitation of FeCl_2_ and FeCl_3_ in alkaline solution in the presence of sodium alginate. Covalent modification methods via alginate have been rarely seen. In order to investigate the effects of the covalent alginate-modified method, alkaloid adsorption experiments were designed to study the properties of alginate-modified Fe_3_O_4_@SiO_2_-NH_2_ nanoparticles.

## 2. Experimental Section

### 2.1. Materials and Physical Measurements

(3-Aminopropyl)trimethoxysilane (APTMS), *N*-Hydroxysuccinimide (NHS) and 1-(3-Dimethylaminopropyl)-3-ethylcarbodiimide hydrochloride (EDC) were purchased from Shanghai source Biological Technology Co., Ltd. (Shanghai, China). Alginate (AA) was purchased from Solarbio Life Science (Beijing Solarbio Biological Technology Co., Ltd., Beijing, China). All commercially available chemicals and solvents were of reagent grade and used without further purification. X-ray powder diffraction (XRD) intensities were measured on a Rigaku D/max-IIIA diffractometer (Cu-Kα, λ = 1.54056 Å). Changes in morphology and size could be characterized by Scanning Electronic Microscopy (SEM) (KAI MEIKE CHEMICAL Co., Ltd., Liaocheng, China). 

XPS spectra were recorded using a Kratos Axis Ultra DLD spectrometer (KAI MEIKE CHEMICAL Co., Ltd.) employing a monochromated Al-Kα X-ray source (hv = 1486.6 eV). The vacuum in the main chamber was kept above 3 × 10^−6^ Pa during XPS data acquisitions. General survey scans (binding energy range: 0–1200 eV; pass energy: 160 eV) and high-resolution spectra (pass energy: 40 eV) in the regions of N1s were recorded. Binding energies were referenced to the C1s binding energy at 284.60 eV.

The adsorption data were obtained by RP-HPLC (Reversed phase high performance liquid chromatography). The HPLC system was from Agilent Technologies 1260 Infinity (Agilent Technologies, SantaClara, CA, USA), and was equipped with a quaternary pump and UV-Vis detector (Agilent Technologies). The chromatographic separation was carried out on an ACE Super C18 column (250 × 4.6 mm i.d., 5 μm, FLM, Guangzhou, China). Mobile phase consisted of 50% solution (*v*/*v*) of acetonitrile in water (0.1% H_3_PO_4_ and 0.1% SDS). The flow rate was 1 mL/min and the column temperature was set to 40 °C. The effluent was monitored at 265 nm and the injection volume was 20 μL. 

### 2.2. Preparation and Modification of Fe_3_O_4_ Nanoparticles

**Figure 1:**
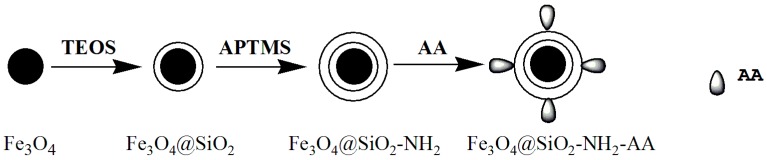
The diagram of surface modification stages.

Magnetite nanoparticles were prepared and modified with TEOS, APTMS, and AA to get Fe_3_O_4_@SiO_2_, Fe_3_O_4_@SiO_2_-NH_2_, and Fe_3_O_4_@SiO_2_-NH_2_-AA nanoparticles, respectively (Figure 1). 

#### 2.2.1. Preparation of Fe_3_O_4_ Nanoparticles

Briefly, 7.5 mL of 0.12 M FeCl_2_ and 7.5 mL of 0.2 M FeCl_3_ solutions were mixed in a 100-mL flask. The whole reaction system was completed under nitrogen protection. After the magnetic stirring was uniform, the reaction system was heated to 55 °C, which maintained for 15 min. 7.2 mL of 3 M NaOH solution was then added to the reaction system. The reaction system was kept at 55 °C for 40 min. Then the reaction system was stirred at 90 °C for 30 min and cooled to room temperature. The black precipitate was collected by magnetic decantation and washed with deionized water repeatedly until the washings were neutral. The obtained black precipitate was then dried over vacuum at 40 °C overnight, which could be used for XRD measurement [14,15].

#### 2.2.2. Preparation of Fe_3_O_4_@SiO_2_

Fe_3_O_4_ (10 mg) was acidized by HCl (0.1 mol/L) under the 100 W of ultrasound for 20 min. The supernatant was discarded after adsorption by the magnet. The residue was washed with ultrapure water for twice, and resuspended in ethanol/ultrapure water (20 mL:5 mL). NH_3_⋅H_2_O (250 μL) was added to the samples of Fe_3_O_4_, and the mixture was reacted for 20 min under the 100 W of ultrasound. TEOS (32 μL) was added into the samples. And then the samples were oscillated at 37 °C and 140 r/min for 6 h, followed by adsorption by the magnet. The supernatant was discarded, and the residue was washed with ethanol for twice to yield Fe_3_O_4_@SiO_2_, which was resuspended in ethanol (4 mL) [16]. 

#### 2.2.3. Preparation of Fe_3_O_4_@SiO_2_-NH_2_

APTMS (50 μL) was dropwise added to the samples of Fe_3_O_4_@SiO_2_ obtained previously, and the mixture was reacted for 24 h. After rinsing with ethanol for twice, the samples named as Fe_3_O_4_@SiO_2_-NH_2_ were vacuum-dried at 80 °C overnight [17]. 

#### 2.2.4. Preparation of Fe_3_O_4_@SiO_2_-NH_2_-AA

An AA solution (5 mg/mL in MES buffer, pH 6.0) was mixed with *N*,*N*-dimethylformamide (DMF; 3:1, *v*/*v*). Then the AA solution (3.75 mg/mL) was converted to *N*-hydroxysuccinimide esters by sequential reaction with EDC (36.3 mg/mL in MES buffer, pH 6.0) for 15 min and NHS (10.95 mg/mL in MES buffer, pH 6.0) for 60 min. The solution was finally introduced to the freshly Fe_3_O_4_@SiO_2_-NH_2_ nanoparticles and reacted overnight at room temperature. After washing by ethanol, the samples of Fe_3_O_4_@SiO_2_-NH_2_-AA could be obtained by vacuum-dried process [18].

### 2.3. Alkaloid Adsorption Test

#### 2.3.1. Preparation of Calibration Standards

100 µg/mL standard solutions in methanol of Palmatine and Berberine were obtained from Solarbio (Beijing, China), and then further diluted in pattern of 1:2 to produce the working solutions with a series of concentrations. The concentration range of calibration standards for Palmatine were 50 µg/mL, 25 µg/mL, 12.5 µg/mL, 6.25 µg/mL, 3.125 µg/mL, 1.5625 µg/mL, 0.78125 µg/mL, while the concentration range of calibration standards for Berberine were 25 µg/mL, 12.5 µg/mL, 6.25 µg/mL, 3.125 µg/mL, 1.5625 µg/mL, 0.78125 µg/mL.

#### 2.3.2. Influence from pH

Approximate 8 mL of mixed standard stock solution (0.5 μg/mL, in methanol, pH 5, 6, 7, 8, 9), 10 mg of Fe_3_O_4_@SiO_2_-NH_2_-AA nanoparticles was ultrasonic shocked for 6 min, and then the supernatant and magnetic nanoparticles were obtained by magnetic separation. The magnetic nanoparticles were washed by deionized water (1 mL × 2). The supernatant and detergent were combined. 1.5 mL of the mixture was dried by nitrogen blower at 80 °C. The residue was redissolved in 400 μL of methanol, which was filtered (0.22 μm) for subsequent HPLC analysis. 

#### 2.3.3. Influence from Adsorption Time

Approximate 8 mL of mixed standard stock solution (0.5 μg/mL, in methanol), 10 mg of Fe_3_O_4_@SiO_2_-NH_2_-AA nanoparticles was ultrasonic shocked for a certain time (2 min, 4 min, 6 min, 8 min, 10 min), and then the supernatant and magnetic particles were obtained by magnetic separation. The magnetic nanoparticles were washed by deionized water (1 mL × 2). The supernatant and detergent were combined. The mixture was dried by nitrogen blower at 80 °C. The residue was redissolved in 400 μL of methanol, which was filtered (0.22 μm) for subsequent HPLC analysis [19,20,21].

## 3. Results and Discussion

### 3.1. XRD Analysis of Fe_3_O_4_ Nanoparticles

**Figure 2:**
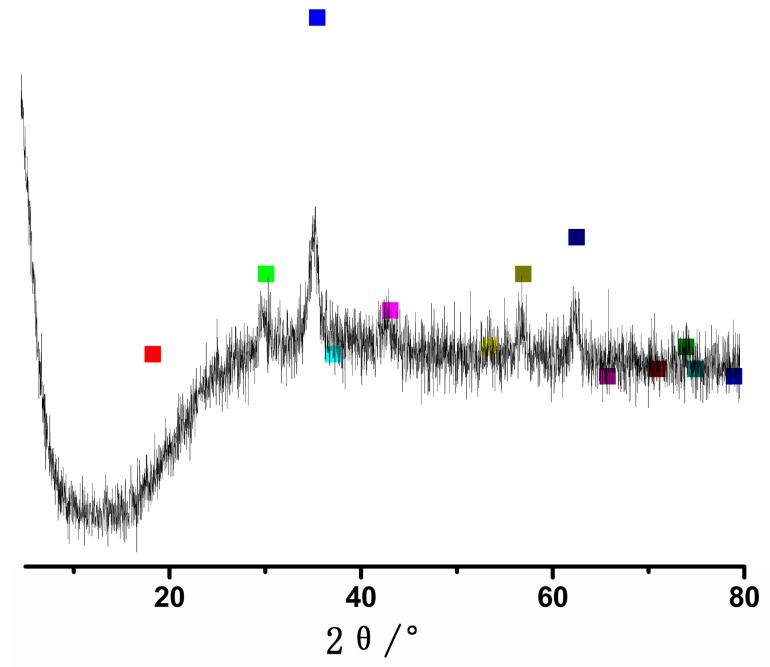
XRD pattern of Fe_3_O_4_ nanoparticles. (Color squares are the standard diffraction spectrum of Fe_3_O_4_).

The XRD pattern of Fe_3_O_4_ nanoparticles is shown in the Figure 2. The peaks at 2θ values of 30.1°, 35.4°, 43.1°, 53.4°, 56.9° and 62.5° are indexed as the diffractions of (220), (311), (222), (422), (511) and (440) respectively, which resembles the standard diffraction spectrum of Fe_3_O_4_ (JCPDSPDF#19-0629) with respect to its reflection peaks positions [5].

### 3.2. FTIR Spectra Analysis of Nanoparticles

**Figure 3:**
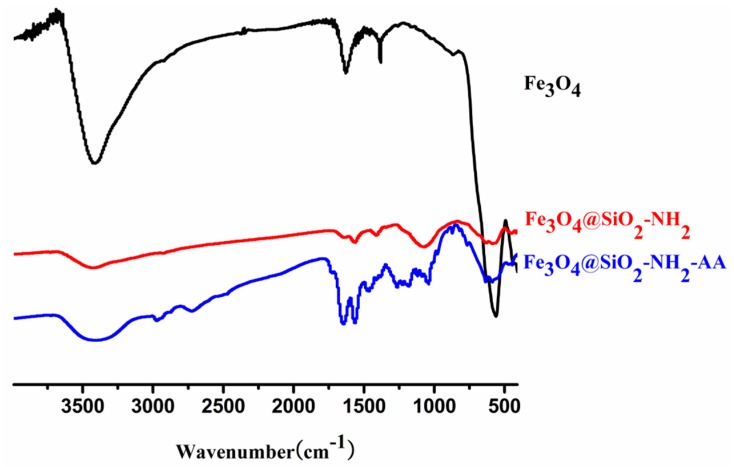
FTIR spectra of: as-prepared Fe_3_O_4_ nanoparticles (black); Fe_3_O_4_@SiO_2_-NH_2_ (red); Fe_3_O_4_@SiO_2_-NH_2_-AA (blue).

The Fe_3_O_4_@SiO_2_-NH_2_ and Fe_3_O_4_@SiO_2_-NH_2_-AA nanoparticles were obtained after the surface modification steps. It is apparent that the IR spectra contains not only the peaks in spectra of Fe_3_O_4_ nanoparticles (Fe-O, 567 cm^−1^) [15]. 1560 cm^−1^ (C-N vibration) reflected that APTMS was successfully modified onto Fe_3_O_4_@SiO_2_nanoparticles [22]. A strong IR peak appears at 1648 cm^−1^, corresponding to the strong bending vibration of the amide I group, which showed that the modification was successful and Fe_3_O_4_@SiO_2_-NH_2_nanoparticles were indeed coated with AA (Figure 3) [17,23,24]. 

### 3.3. XPS Analysis of Nanoparticles

**Figure 4:**
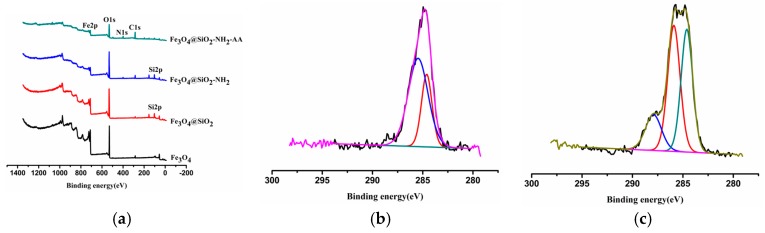
(**a**) XPS wide scan spectra of Fe_3_O_4_, Fe_3_O_4_@SiO_2_, Fe_3_O_4_@SiO_2_-NH_2_, Fe_3_O_4_@SiO_2_-NH_2_-AA nanoparticles; (**b**) High-resolution XPS C1s spectra of Fe_3_O_4_@SiO_2_-NH_2_; (**c**) High-resolution XPS C1s spectra of Fe_3_O_4_@SiO_2_-NH_2_-AA.

Figure 4a shows the low-resolution XPS survey spectra of Fe_3_O_4_, Fe_3_O_4_@SiO_2_, Fe_3_O_4_@SiO_2_-NH_2_ and Fe_3_O_4_@SiO_2_-NH_2_-FA samples, all of which are semiquantitative. The low-resolution XPS survey spectra (Figure 4a) of Fe_3_O_4_@SiO_2_-NH_2_ have peaks of N1s, which showed that APTMS have been modified successfully. High-resolution C1s XPS spectra of the Fe_3_O_4_@SiO_2_-NH_2_ samples have peaks at 284.603 eV (C-H/C-C) and 285.459 eV (C-O/C-N) (Figure 4b). High-resolution C1s XPS spectra of the Fe_3_O_4_@SiO_2_-NH_2_-AA samples have peaks at 284.605 eV (C-H/C-C), 285.891 eV (C-O/C-N), and 287.916 eV (O-C=O/O=C-NH) (Figure 4c), which showed that amide reaction was successful [25]. 

### 3.4. SEM Analysis of Nanoparticles

**Figure 5:**
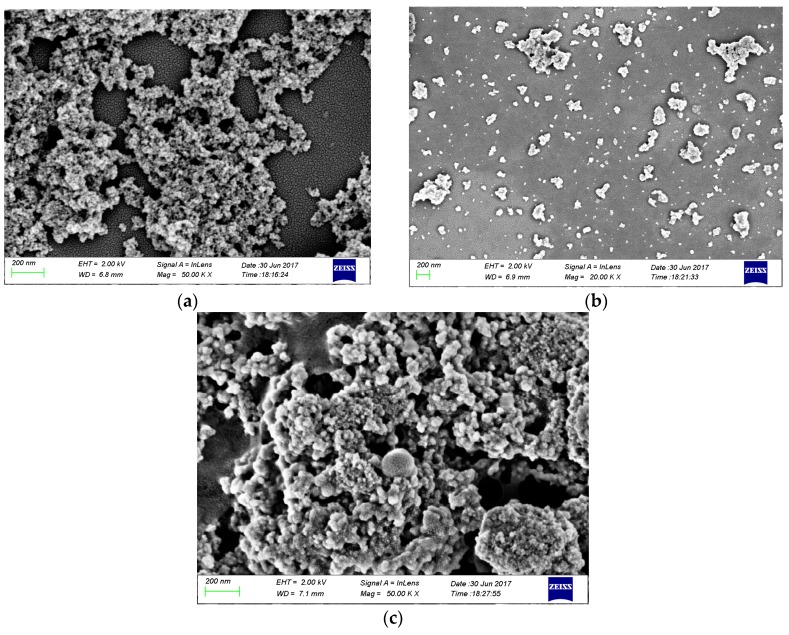
The SEM images of nanoparticles: (**a**) Fe_3_O_4_, (**b**) Fe_3_O_4_@SiO_2_-NH_2_, (**c**) Fe_3_O_4_@SiO_2_-NH_2_-AA.

Figure 5a–c show SEM images of Fe_3_O_4_, Fe_3_O_4_@SiO_2_-NH_2_, and Fe_3_O_4_@SiO_2_-NH_2_-AA nanoparticles. Small particle size of Fe_3_O_4_ particles is obvious, while a good dispersion effect could be achieved by Fe_3_O_4_@SiO_2_-NH_2_ nanoparticles. As for Fe_3_O_4_@SiO_2_-NH_2_-AA nanoparticles, no good dispersion could be achieved, while better morphology could be achieved, which showed that AA was successfully modified onto Fe_3_O_4_@SiO_2_-NH_2_ nanoparticles [22]. Almost all particle size of Fe_3_O_4_ particlesis below 100 nm, as for Fe_3_O_4_@SiO_2_-NH_2_ nanoparticles and Fe_3_O_4_@SiO_2_-NH_2_-AA nanoparticles, particle size is becoming larger and larger, which could also prove that the modification is successful. 

### 3.5. Analysis of Alkaloid Adsorption Test

Electrostatic interactions between alkaloids and charged surfaces, therefore, often play a major role in the adsorption behavior of alkaloids. Therefore, Palmatine and Berberine were selected for alkaloid adsorption assay in the current study. 

**Figure 6:**
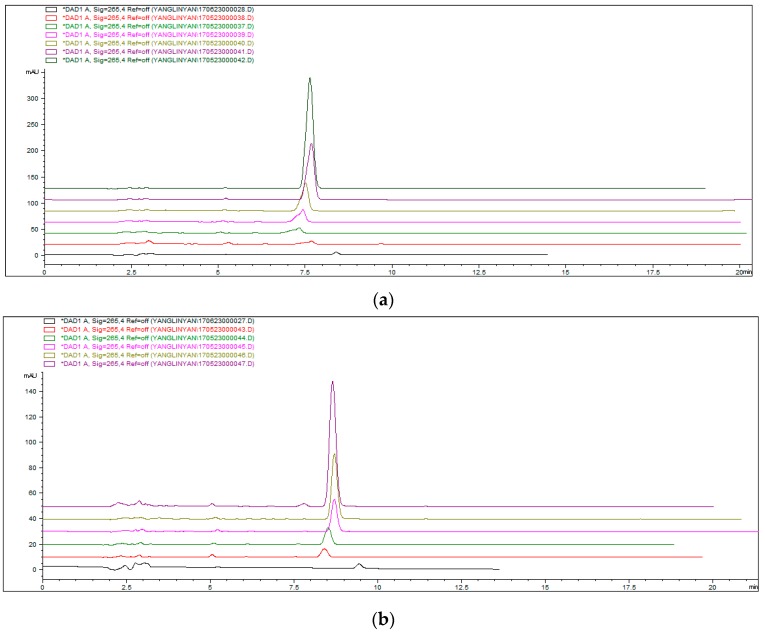

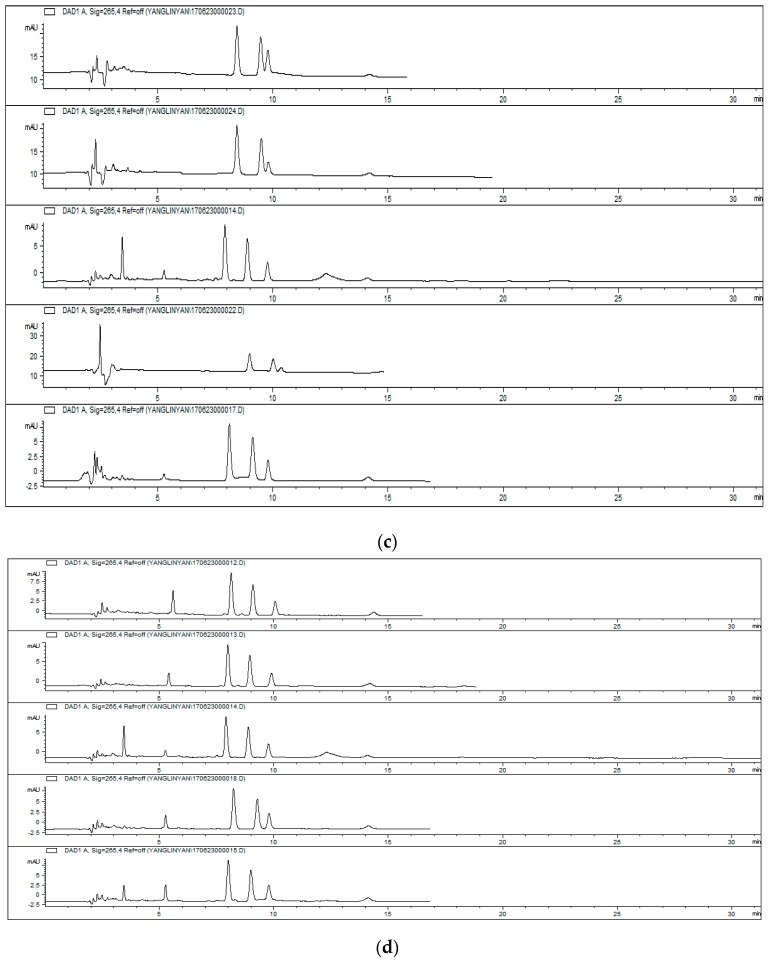
(**a**) Concentration gradient chromatogram for Palmatine. (Standrad curve: y = 5.41437 + 61.51865x, *R* = 0.99958, linear range: 0.78125–50 μg/mL); (**b**) Concentration gradient chromatogram for Berberine. (Standard curve: y = −3.38806 + 53.63054x, *R* = 0.99899, linear range: 0.78125–25 μg/mL); (**c**) HPLC charomatograms of the supernatant after adsorption. Conditions: pH adjustment was as follows: 5, 6, 7, 8, 9; adsorption time was 6 min; (**d**) HPLC charomatograms of the supernatant after adsorption. Conditions: adsorption time adjustment was as follows: 2 min, 4 min, 6 min, 8 min, 10 min, while pH 7 was used.

Figure 6a is the chromatogram associated with the concentrations of the standard curve, which belongs to Palmatine. Figure 6b is the chromatogram associated with the concentrations of the standard curve, which belongs to Berberine. The Equation process is as follows:

(1) $$\frac{CV_{2}}{V_{1}}\times V_{0}=m$$

(2) $$Ap=\frac{C_{0}V-m}{C_{0}V}$$

V_0_ = 10 mL, V_1_ = 1.5 mL, V_2_ = 0.4 mL, m is the capacity of alkaloid in the supernatant and detergent, C is the concentration of the supernatant and detergent, which could be obtained by the standard curve.

C_0_ = 0.5 μg/mL, V = 8 mL, Ap is the adsorption percentage of alkaloid. 

From Table S1, as for Palmatine and Berberine, the most adsorption could be obtained at pH 8. Considering the effect of alkaline on liquid phase system, the adsorption conditions of pH 8 has been chosen. The adsorption percentage of Palmatine was 22.2%, and the adsorption percentage of Berberine was 23.6%. At pH 8, the carboxylic acid of Fe_3_O_4_@SiO_2_-NH_2_-AA nanoparticles was converted to a negatively-charged carboxylate ion. Therefore, quaternary ammonium alkaloids were significantly adsorbed onto the carboxylic acid-rich surface, possibly due to electrostatic interactions. The results from this study seem to fit well with a previous report on the study of the charge interaction of alkaloids and polyelectrolyte films.

From Table S2, as for Berberine, the most adsorption could be obtained at 8 min. While the most adsorption could be obtained at 10 min for Palmatine. Considering the effect of adsorption time on liquid phase system, the adsorption conditions of 8 min has been chosen. The adsorption percentage of Palmatine was 8.67%, and the adsorption percentage of Berberine was 7.25%.

The effect of pH was greater than that of adsorption time. Considering the above conditions, pH 8 and the adsorption time of 8 min could be chosen for further uses. 

## 4. Conclusions

In conclusion, magnetite (Fe_3_O_4_) could be prepared by FeCl_2_ and FeCl_3_, which is a ferromagnetic black color iron oxide of both Fe(II) and Fe(III). XRD was used for the determination of Fe_3_O_4_ nanoparticles. The peaks at 2θ values of 30.1°, 35.4°, 43.1°, 53.4°, 56.9° and 62.5° resemble the standard diffraction spectrum of Fe_3_O_4_ (JCPDSPDF#19-0629) with respect to its reflection peaks positions. Fe_3_O_4_ could be used for modification at the subsequent trials. Fe_3_O_4_@SiO_2_ nanoparticles were successfully obtained by TEOS. Fe_3_O_4_@SiO_2_-NH_2_ nanoparticles were prepared by APTMS, while Fe_3_O_4_@SiO_2_-NH_2_-AA nanoparticles were obtained by activated AA via amidation reaction. IR, XPS and SEM analysis were used for the characterizations of Fe_3_O_4_@SiO_2_-NH_2_ and Fe_3_O_4_@SiO_2_-NH_2_-AA nanoparticles. Alkaloid adsorption experiments implied that Fe_3_O_4_@SiO_2_-NH_2_-AA nanoparticles as a absorbent could be used for the adsorption of the alkaloids. At pH 8, the carboxylic acid of Fe_3_O_4_@SiO_2_-NH_2_-AA nanoparticleswas converted to a negatively-charged carboxylate ion. Therefore, quaternary ammonium alkaloids were significantly adsorbed onto the carboxylic acid-rich surface, possibly due to electrostatic interactions. As for Palmatine and Berberine, the most adsorption could be obtained at pH 8 when the adsorption time was 6 min. The adsorption percentage of Palmatine was 22.2%, while the adsorption percentage of Berberine was 23.6% at pH 8. As for the effect of adsorption time on liquid phase system, the adsorption conditions of 8 min has been chosen when pH 7 was used. Considering the above conditions, pH 8 and the adsorption time of 8 min could be chosen for further uses. This work demonstrates the potential of AA modification in a Fe_3_O_4_-based alkaloid adsorption study. In further experiments, when the amidation reaction is performed, residual carboxyl groups from AA on the modified Fe_3_O_4_@SiO_2_-NH_2_-AA nanoparticles may be used for bio-molecule immobilization.

## Supplementary Materials

The supplementary materials are available online.
