# Supplementary material for: Modification and Characterization of Fe3O4 Nanoparticles for Use in Adsorption of Alkaloids
Source: Molecules. 2018 Mar 2;23(3):562. doi: 10.3390/molecules23030562 (PMC6017166; doi:10.3390/molecules23030562)
Supplement: Supplementary file 1 [file molecules-23-00562-s001.pdf]

Supporting Information

Modification and Characterization of Fe_3O_4 Nanoparticles for Use in Adsorption of Alkaloids

Linyan Yang [1,2], Jing Tian [1], Jiali Meng [1], Ruili Zhao [1], Cun Li [1,*], Jifei Ma [1,*], Tianming Jin [1,*]

[1]College of Animal Science and Veterinary Medicine, Tianjin Agricultural University, Tianjin, 300384, China

[2] Guangxi Key Laboratory for the Chemistry and Molecular Engineering of Medicinal Resources, Chemical and pharmaceutical college of Guangxi Normal University, Guilin 541004, China

Table S1 Retention time and peak area of alkaloids when HPLC conditions were adjusted as follows: pH range was from 5 to 9; adsorption time was 6min.

Conditions	Retention file	Retention time	Peak area
pH5, 6min	170623000023.D	8.418min	97.7
		9.451min	83.1
pH6, 6min	170623000024.D	8.417min	97.5
		9.476min	80.3
pH7, 6min	170623000014.D	7.899min	88.8
		8.87min	76.6
pH8, 6min	170623000022.D	8.968min	77.2
		9.994min	58.1
pH9, 6min	170623000017.D	8.086min	84.9
		9.101min	70.0

Table S2 Retention time and peak area of alkaloids when HPLC conditions were adjusted as follows: adsorption time was from 2min to 10min, while pH7 was used.

Conditions	Retention file	Retention time	Peak area
pH7, 2min	170623000012.D	8.12min	90.8
		9.07min	74.3
pH7, 4min	170623000013.D	7.975min	93.1
		8.935min	78.6
pH7, 6min	170623000014.D	7.899min	88.8
		8.87min	76.6
pH7, 8min	170623000018.D	8.229min	89.7
		9.263min	71.4
pH7, 10min	170623000015.D	7.994min	87.8
		8.986min	74.9

*Correspondence Author. Fax: +86-22-23781297. Tel: 13920589691, 13920081772, 13114881898.

E-mail address: hhlicun@163.com, hbmjfts@126.com, JTMSCI@163.com.
